# Supplementary material for: Identification of HIV transmitting CD11c+ human epidermal dendritic cells
Source: Nat Commun. 2019 Jun 21;10:2759. doi: 10.1038/s41467-019-10697-w (PMC6588576; doi:10.1038/s41467-019-10697-w)
Supplement: Supplementary file 1 — Supplementary information [file 41467_2019_10697_MOESM1_ESM.pdf]

Identification of HIV Transmitting CD11c^{+} Human Epidermal Dendritic Cells

Bertram et al.,

Supplemental Figures

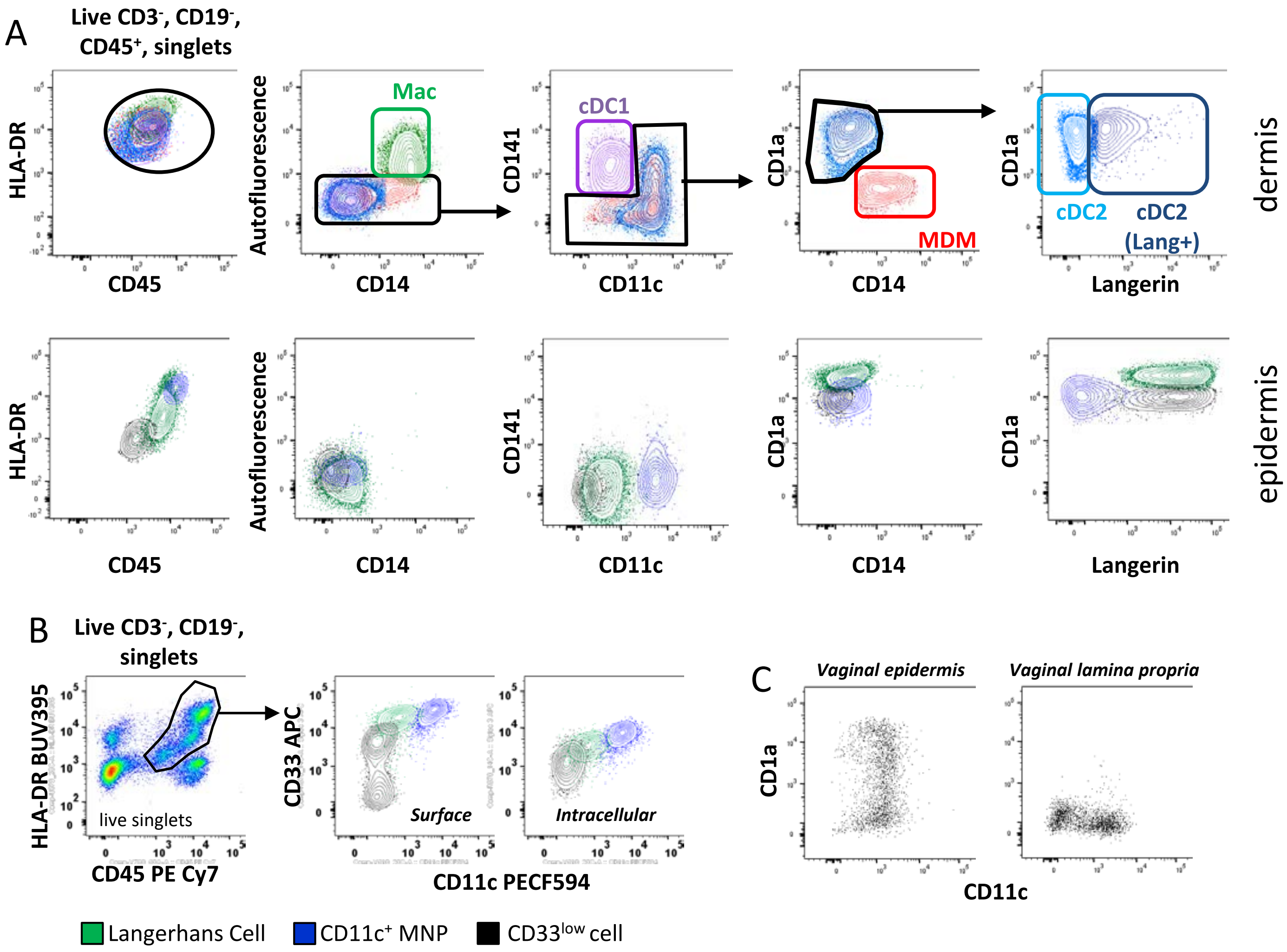

Supplementary Figure 1: Abdominal dermal and epidermal, and vaginal lamina propria and epidermal cells were liberated using Type IV collagenase and cells were stained for flow cytometry as follows. **A)** Abdominal dermal and epidermal cells were stained for live/dead, HLA-DR, CD45, CD3, CD19 and CD14. An FMO control was included for CD14. Results are representative of at least 75 donors. **B)** Surface and intracellular staining was carried out on abdominal epidermal cells for live/dead, HLADR, CD45, CD3, CD19, CD33 and CD11c. Results are representative of 3 donors. **C)** Vaginal lamina propria and epidermal cells were stained for live/dead, HLA-DR, CD45, CD3, CD19, CD11c and CD1a. Results are representative of 5 donors.

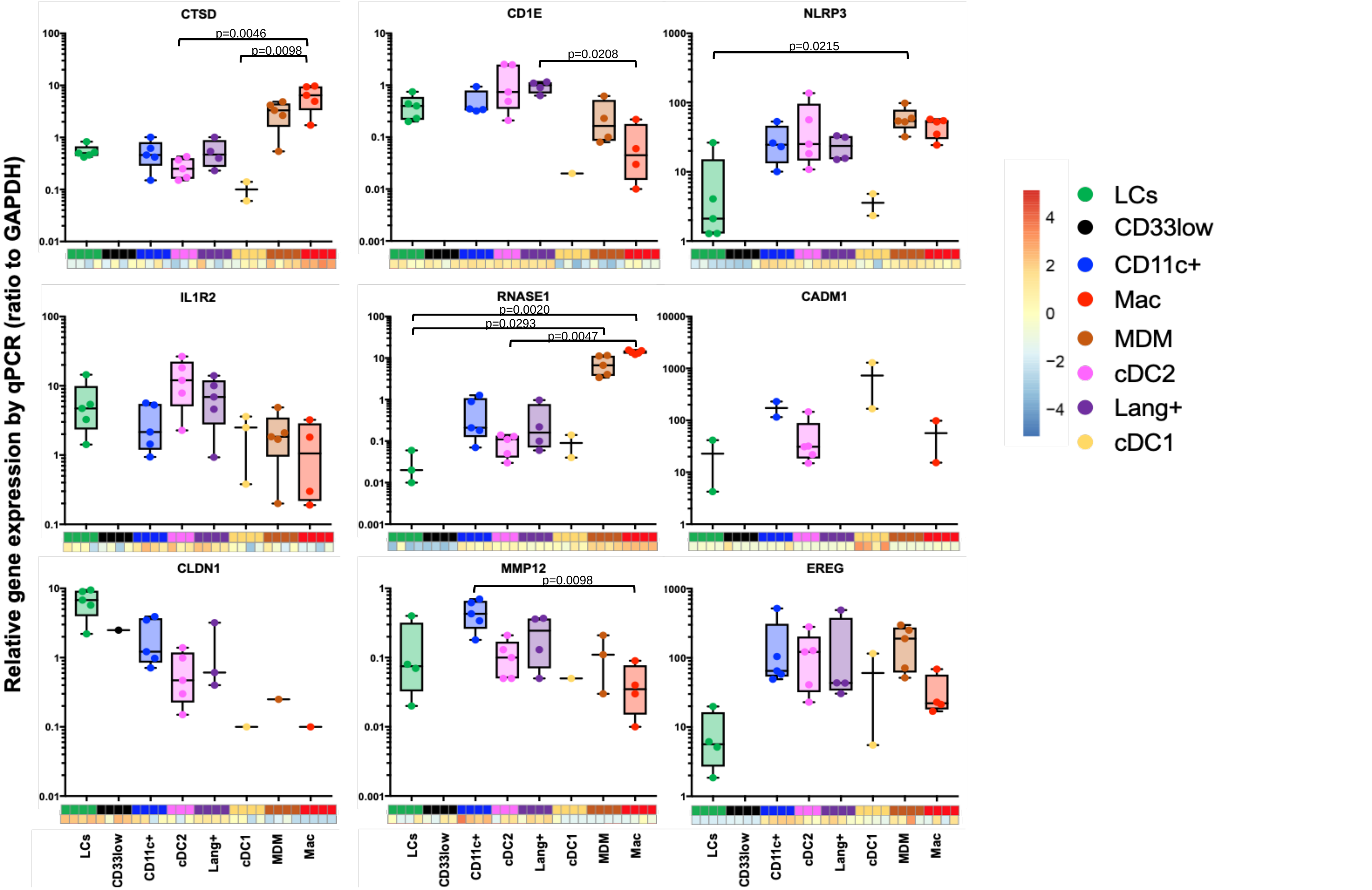

Supplementary Figure 2: ***Confirmation of RNAseq clustering by QPCR.*** Epidermal LCs, CD11c^{+}DCs and dermal cDC1, cDC2 (langerin^{+} and $^{-}$), monocyte-derived macrophages and tissue resident macrophages were FACS sorted from 5 individual donors. RNA was extracted and reverse transcribed to cDNA. Nine genes that uniquely identified each cluster in the heatmaps shown in Figure 2 were tested by QPCR to confirm RNAseq analysis. The cut-off values were plotted as a box and whisker plots plot representing, the upper and lower quartile, the central bar represents the median, while the whiskers show the minimum and maximum for each sample, with each dot representing an individual donor. The heatmaps from the RNAseq clustering analysis are shown below each graph.

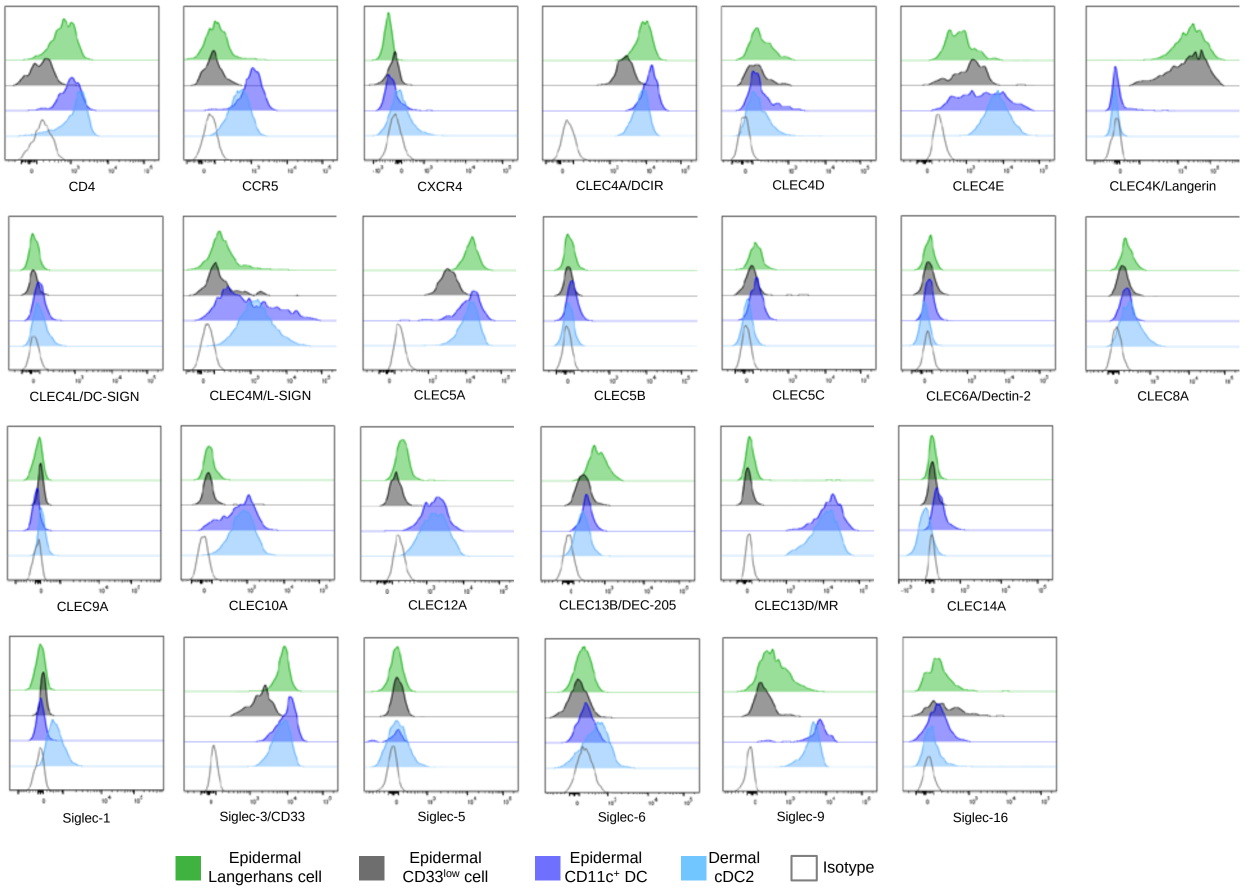

Supplementary Figure 3: Ex vivo epidermal MNPs and dermal cDC2 were isolated from human skin using either Blend F or Type IV collagenase and the surface expression was determined for a range of pathogen binding receptors and HIV entry receptors by flow cytometry. Representative histograms for each marker on each cell type are shown (n = 5).

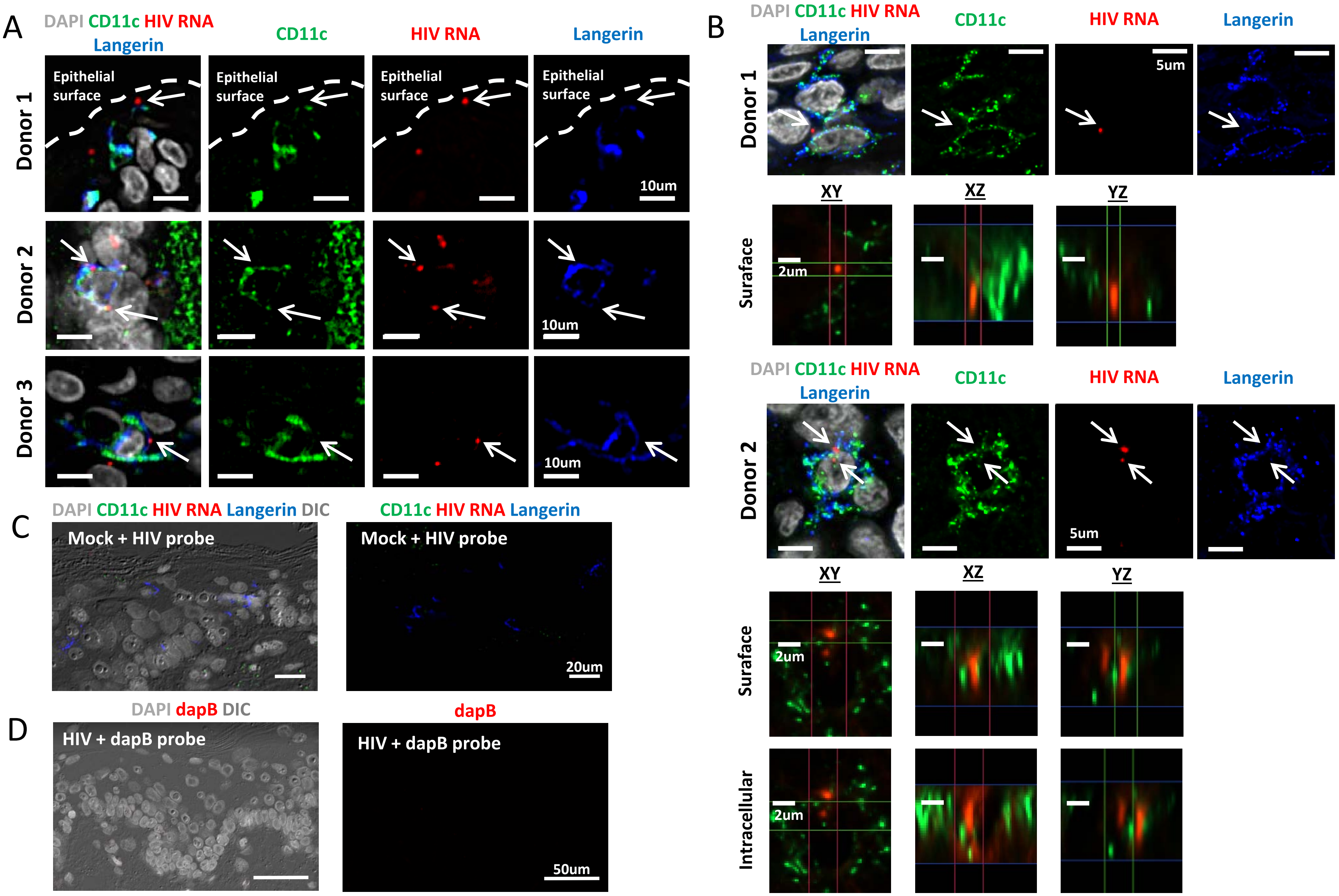

Supplementary Figure 4: Human foreskin explants were treated with HIV_{BaL} or transmitted/founder HIV_{Z3678M} or PBS (mock) for 2-3 hours before being fixed and paraffin embedded. The tissue was then sectioned and probed for HIV RNA using RNAscope coupled with immunofluorescent staining for CD11c and Langerin. **A)** Epidermal $CD11c^{+}$ DCs from three separate donors interacting with HIV (white arrows). **B)** Epidermal CD11c+ DCs interacting with transmitted/founder HIV_{Z3678M} in two separate donors (white arrows). Z- stacks were performed with 0.1 micron Z spacing and XY, XZ and YZ projections are shown. **C)** Mock treated tissue probed for HIV RNA and stained for CD11c and langerin. Representative of n=5. **D)** HIV treated tissue probed for dapB (negative probe control). Representative of n=5.
