# Supplementary material for: Identification of HIV transmitting CD11c+ human epidermal dendritic cells
Source: Nat Commun. 2019 Jun 21;10:2759. doi: 10.1038/s41467-019-10697-w (PMC6588576; doi:10.1038/s41467-019-10697-w)
Supplement: Supplementary file 3 — Reporting Summary [file 41467_2019_10697_MOESM3_ESM.pdf]

nature research

Corresponding author(s): Andrew Harman

Last updated by author(s): May 10, 2019

Reporting Summary

Nature Research wishes to improve the reproducibility of the work that we publish. This form provides structure for consistency and transparency in reporting. For further information on Nature Research policies, see Authors & Referees and the Editorial Policy Checklist.

Statistics

For all statistical analyses, confirm that the following items are present in the figure legend, table legend, main text, or Methods section.

n/a	Confirmed	
☐	☒	The exact sample size (*n*) for each experimental group/condition, given as a discrete number and unit of measurement
☐	☒	A statement on whether measurements were taken from distinct samples or whether the same sample was measured repeatedly
☐	☒	The statistical test(s) used AND whether they are one- or two-sided<br>*Only common tests should be described solely by name; describe more complex techniques in the Methods section.*
☒	☐	A description of all covariates tested
☐	☒	A description of any assumptions or corrections, such as tests of normality and adjustment for multiple comparisons
☐	☒	A full description of the statistical parameters including central tendency (e.g. means) or other basic estimates (e.g. regression coefficient) AND variation (e.g. standard deviation) or associated estimates of uncertainty (e.g. confidence intervals)
☐	☒	For null hypothesis testing, the test statistic (e.g. *F*, *t*, *r*) with confidence intervals, effect sizes, degrees of freedom and *P* value noted<br>*Give P values as exact values whenever suitable.*
☒	☐	For Bayesian analysis, information on the choice of priors and Markov chain Monte Carlo settings
☒	☐	For hierarchical and complex designs, identification of the appropriate level for tests and full reporting of outcomes
☐	☐	Estimates of effect sizes (e.g. Cohen's *d*, Pearson's *r*), indicating how they were calculated

Our web collection on statistics for biologists contains articles on many of the points above.

Software and code

Policy information about availability of computer code

Data collection	flow cytometry: BD DIVA<br>TZMBL reading: AID ELPISPOT Reader
Data analysis	Flowjo, Fiji (Madison version), Prism - graph pad

For manuscripts utilizing custom algorithms or software that are central to the research but not yet described in published literature, software must be made available to editors/reviewers. We strongly encourage code deposition in a community repository (e.g. GitHub). See the Nature Research guidelines for submitting code & software for further information.

Data

Policy information about availability of data

All manuscripts must include a data availability statement. This statement should provide the following information, where applicable:

- Accession codes, unique identifiers, or web links for publicly available datasets
- A list of figures that have associated raw data
- A description of any restrictions on data availability

Figure 2: The RNA sequencing data have been deposited in the Gene Expression Omnibus (GEO) database under accession code: GSE130804.

Field-specific reporting

Please select the one below that is the best fit for your research. If you are not sure, read the appropriate sections before making your selection.

☒ Life sciences ☐ Behavioural & social sciences ☐ Ecological, evolutionary & environmental sciences

Life sciences study design

All studies must disclose on these points even when the disclosure is negative.

Sample size	The sample sizes were not predetermined. For all key experiments at least three donors were analysed. Additional donors were used where possible.
Data exclusions	No data were excluded from the analysis
Replication	All findings were reliably reproducible
Randomization	No randomization was necessary
Blinding	No blinding was necessary

Reporting for specific materials, systems and methods

We require information from authors about some types of materials, experimental systems and methods used in many studies. Here, indicate whether each material, system or method listed is relevant to your study. If you are not sure if a list item applies to your research, read the appropriate section before selecting a response.

Materials & experimental systems

n/a	Involved in the study	
☐	☒	Antibodies
☐	☒	Eukaryotic cell lines
☒	☐	Palaeontology
☒	☐	Animals and other organisms
☐	☒	Human research participants
☒	☐	Clinical data

Methods

n/a	Involved in the study	
☒	☐	ChIP-seq
☐	☒	Flow cytometry
☒	☐	MRI-based neuroimaging

Antibodies

Antibodies used	All antibodies (including clone number) are described in the methods.
Validation	All antibodies were comercially available, specificity had been described by the manufacturer

Eukaryotic cell lines

Policy information about cell lines

Cell line source(s)	JLTR - NIH AIDS repository (Cat# 11586)<br>TZMBL1 - NIH AIDS repository (Cat# 100)
Authentication	Both cells are only infectible with HIV due their high CCR5 expression which was regularly checked by flow cytometry. TZMBL1 cells could be distinguished from JLTR cells as they express beta galactosidase when infected whereas JLTR cells express GFP.
Mycoplasma contamination	All cell lines were tested and shown to be free from mycoplasma contamination
Commonly misidentified lines<br>(See ICLAC register)	Neither JLTR or TZMBL1 cells are listed in the most recent ICLAC database of commonly misidentified cell lines.

Human research participants

Policy information about studies involving human research participants

Population characteristics	Healthy human abdominal and genital tissues were obtained within 30 minutes of surgery from a range of patients undergoing plastic surgery (abdomen, labia, vagina), circumcision (foreskin), gender reassignment (glans penis, fossa navicularis) or colorectal surgery (anal canal, anal verge) with written consent from all donors. Patients were all aged between 18-71 and all tissue was obtained in a deidentified fashion.
Recruitment	Tissue from surgery that was otherwise discarded was used for this study. Surgeons asked for consent at consult.

Ethics oversight	This study was approved by the Western Sydney Local Area Health District (WSLHD) Human Research Ethics Committee (HREC); reference number (4192) AU RED HREC /15 WMEAD/11.

Note that full information on the approval of the study protocol must also be provided in the manuscript.

Flow Cytometry

Plots

Confirm that:

☒ The axis labels state the marker and fluorochrome used (e.g. CD4-FITC).

☒ The axis scales are clearly visible. Include numbers along axes only for bottom left plot of group (a 'group' is an analysis of identical markers).

☒ All plots are contour plots with outliers or pseudocolor plots.

☒ A numerical value for number of cells or percentage (with statistics) is provided.

Methodology

Sample preparation	Thoroughly described in manuscript
Instrument	BD Fortessa, BD CantoII (transfer assays only), BD Influx (sort)
Software	Collect: BD DIVA<br>Analyse: FlowJo V10
Cell population abundance	Thoroughly described in manuscript
Gating strategy	Thoroughly described in manuscript

☒ Tick this box to confirm that a figure exemplifying the gating strategy is provided in the Supplementary Information.
